# Automated contouring of CTV and OARs in planning CT scans using novel hybrid convolution-transformer networks for prostate cancer radiotherapy

**DOI:** 10.1007/s12672-024-01177-9

**Published:** 2024-07-31

**Authors:** Najmeh Arjmandi, Shahrokh Nasseri, Mehdi Momennezhad, Alireza Mehdizadeh, Sare Hosseini, Shokoufeh Mohebbi, Amin Amiri Tehranizadeh, Zohreh Pishevar

**Affiliations:** 1 Department of Medical Physics, Faculty of Medicine, Mashhad University of Medical Sciences, Mashhad, Iran; 2 Medical Physics Research Center, Faculty of Medicine, Mashhad University of Medical Sciences, Mashhad, Iran; 3 Ionizing and Non-Ionizing Radiation Protection Research Center, School of Paramedical Sciences, Shiraz University of Medical Sciences, Shiraz, Iran; 4 Department of Radiation Oncology, Mashhad University of Medical Sciences, Mashhad, Iran; 5 Cancer Research Center, Mashhad University of Medical Sciences, Mashhad, Iran; 6 Medical Physics Department, Reza Radiation Oncology Center, Mashhad, Iran; 7 Department of Medical Informatics, Faculty of Medicine, Mashhad University of Medical Sciences, Mashhad, Iran

**Keywords:** Deep learning, Male pelvic radiotherapy, Prostate segmentation, Vision transformer, Convolutional neural network, CT images

## Abstract

**Purpose objective(s):**

Manual contouring of the prostate region in planning computed tomography (CT) images is a challenging task due to factors such as low contrast in soft tissues, inter- and intra-observer variability, and variations in organ size and shape. Consequently, the use of automated contouring methods can offer significant advantages. In this study, we aimed to investigate automated male pelvic multi-organ contouring in multi-center planning CT images using a hybrid convolutional neural network-vision transformer (CNN-ViT) that combines convolutional and ViT techniques.

**Materials/methods:**

We used retrospective data from 104 localized prostate cancer patients, with delineations of the clinical target volume (CTV) and critical organs at risk (OAR) for external beam radiotherapy. We introduced a novel attention-based fusion module that merges detailed features extracted through convolution with the global features obtained through the ViT.

**Results:**

The average dice similarity coefficients (DSCs) achieved by VGG16-UNet-ViT for the prostate, bladder, rectum, right femoral head (RFH), and left femoral head (LFH) were 91.75%, 95.32%, 87.00%, 96.30%, and 96.34%, respectively. Experiments conducted on multi-center planning CT images indicate that combining the ViT structure with the CNN network resulted in superior performance for all organs compared to pure CNN and transformer architectures. Furthermore, the proposed method achieves more precise contours compared to state-of-the-art techniques.

**Conclusion:**

Results demonstrate that integrating ViT into CNN architectures significantly improves segmentation performance. These results show promise as a reliable and efficient tool to facilitate prostate radiotherapy treatment planning.

## Introduction

Accurate contouring of both the clinical target volume (CTV) and critical organs at risk (OARs) including bladder, rectum, right femoral head (RFH), and left femoral head (LFH) is crucial for successful radiation therapy of prostate cancer [1, 2]. Manual contouring of the prostate region in CT images is a time-consuming process [3, 4]. This issue can result in delays in starting radiotherapy treatment, particularly in clinics with limited resources [5]. Reports have demonstrated significant variations in contouring results among different experts [6]. Furthermore, the low contrast of soft tissue in male pelvic CT images often leads to unclear boundaries between the prostate region and surrounding organs [7], thereby rendering accurate contouring a challenging endeavor. Additional complexities arise from the considerable variability in the shapes and size of male pelvic organs [7, 8].

To address these challenges, many automatic contouring methods have been proposed. Ling Ma et al. [9] conducted a hybrid approach that combined deep learning techniques with an Atlas model to automatically contour the prostate on 2D CT images. They obtained preliminary contour using a convolutional neural network (CNN) and subsequently refined the CNN-derived results with the atlas method. This proposed method yielded a dice similarity coefficient (DSC) of 86.8%.

Kazemifar et al. [8] developed an automatic approach to contour the prostate, rectum, bladder, and femoral heads in CT images. They designed a 2D U-Net that received CT images slice-by-slice and outputted the corresponding segmented image. In another study [10], they used a 2D U-Net for organ localization and then used a 3D U-Net approach to achieve precise contouring. The combination of the 2D localization network and a 3D contouring network led to an improvement in the Dice similarity coefficient for the prostate, increasing from 88 to 90%.

He et al. [11] developed a two-step framework for CT prostate segmentation using fully convolutional networks. The first stage localizes the prostate region, while the second stage precisely segments it using a multi-task U-Net architecture. The proposed network uses voxel-wise sampling in a multitask learning module, enhancing the quality of the learned feature space.

Wang et al. [12] introduced an automatic deep learning-based prostate segmentation method for 313 CT male pelvic scans. Their segmentation framework includes an organ localization model, a boundary-sensitive representation model, and a multi-label cross-entropy loss function. This approach outperforms baseline fully convolutional networks.

Pan et al. proposed a token-based transformer network for multi-organ segmentation using CT images. Their hybrid architecture combines a ResNet-like encoder, a transformer module for capturing global dependencies, and a mirroring decoder for detailed segmentations. The network's performance was evaluated using several metrics. Dice scores for the prostate, rectum, bladder, left femoral head, and right femoral head reached 0.84, 0.89, 0.94, 0.95, and 0.95, respectively. Hausdorff distances ranged from 2.56 mm to 6.59 mm, while mean surface distances varied from 0.91 mm to 4.97 mm, and residual mean square distances from 1.24 mm to 2.03 mm [13].

Kawula et al. investigated the efficacy of a 3D U-Net model for segmenting the prostate, bladder, and rectum in CT images. Geometric accuracy was assessed using the DSC and 95% HD. The DSC values for the prostate, bladder, and rectum were 0.87, 0.97, and 0.89, respectively. The average and 95% HD for these organs were all below 1.6 mm and 4 mm, below 0.95 mm and 2.5 mm, and below 1.4 mm and 5 mm, respectively [14].

Shen et al. proposed a convolutional CUNet network for automated contouring of the CTV and OARs in prostate cancer radiotherapy. CUNet leverages a 3D U-Net architecture with an attention center block that enhances feature refinement and performance by selectively emphasizing informative features while suppressing less relevant ones. The model's performance was evaluated using Dice Similarity Coefficient (DSC) and 95th percentile Hausdorff distance (95HD) metrics for CTV and OAR delineation. The mean DSC and 95HD values for the defined CTVs were (0.84 ± 0.05) and (5.04 ± 2.15) mm, respectively. For OARs, the DSC values ranged from 0.783 to 0.913, with corresponding 95HD values spanning 1.424 to 6.278 mm [15].

Mofid et al. investigated the use of a 3D nnU-net architecture for automatic segmentation in prostate cancer patients. The nnU-net architecture adheres to a 3D U-Net pattern, incorporating an encoder-decoder structure with skip connections. The algorithm demonstrated high performance, achieving DSC of 0.97 (bladder), 0.96 (right femur head), 0.9 (rectum), 0.82 (prostate), 0.77 (lymph nodes), and 0.69 (seminal vesicles). Corresponding HD were 4.13, 3.58, 10.04, 3.68, 15.5, and 10.95 mm, respectively [16].

Although these studies have demonstrated promising results achieved by CNNs in male pelvic multi-organ contouring, the precise delineation of the prostate region on CT images using CNNs still remains a challenging task. One notable drawback of employing CNNs for medical image segmentation is their limited ability to capture global dependencies [17]. CNNs typically have localized receptive fields, which means they focus on small regions of the input image at a time. In medical imaging, where global context and spatial relationships are critical for accurate segmentation, this limitation can negatively impact the performance of CNN-based models [18].

Vision transformer involves applying transformer-based models, which have shown great success in natural language processing tasks, to the task of segmenting medical images [18, 19]. Unlike CNNs, ViTs operate on the entire image rather than localized regions. This allows them to capture long-range dependencies and contextual information across the image [18, 20]. However, directly applying up-sampling techniques on ViTs is ineffective in adequately restoring fine-grained information, often resulting in a coarse segmentation outcome [18].

Many studies have focused on the hybrid CNN-ViT architecture [18, 20] to maximize the advantages offered by both models. In TransUnet [18], the feature tensor obtained from the ViT was used and combined with the hierarchical deconvolved features of matching resolution from the CNN in the decoder module.

This study proposes the use of hybrid CNN-ViT networks for the male pelvic multi-organ contouring of prostate cancer patients. In this paper, we implemented a novel approach that combines the ViT and CNN architectures to capture detailed features with long-range dependency capabilities. Our main objective was to propose and employ an attention-based fusion mechanism to merge the detailed features extracted through the convolutional model with the global features obtained through the transformer model. We used 104 radiotherapy planning CT volumes to train and evaluate two CNN and two hybrid CNN-ViT networks.

## Materials and methods

### Patient data

For this study, we used retrospective data from 104 localized prostate cancer patients. An attending radiation oncologist delineated the target organ (prostate) and critical OARs (bladder, rectum, RFH, and LFH) on CT images using ISOgray radiotherapy treatment planning software (DOSIsoft, SA, France).

Data collection for this study involved multiple centers where CT images were acquired using different scanners from various manufacturers. Research data were collected from three radiotherapy centers in Mashhad, Iran: the Research and Treatment Center of Imam Reza Hospital, Reza Radiotherapy and Oncology Center, and Razavi Hospital. Radiotherapy planning CT images of prostate cancer patients were obtained using different CT scanners, including NeuVis (PNMS manufacturer), LightSpeed (GE manufacturer), and Somatom Sensation Open (Siemens manufacturer).

### Data preprocessing

The planning CT images were cropped to exclude the non-pelvic regions, as they lack any relevant information for network training and only contribute to increased computational time. Data normalization and standardization were then applied, rescaling the images to have a mean of 0 and a standard deviation (SD) of 1. Finally, the pre-processed CT images were shuffled and split into three sets: 70% for training, 10% for validation, and 20% for testing. To enhance data variability, we applied online data augmentation techniques to the training set. This approach, generating augmented data dynamically during training, offers flexibility, and minimizes storage needs. Augmentation techniques included rotations, flipping (horizontal and vertical), cropping, shifting, zooming, random local rotations, and shearing.

### ViT model implementation

The research procedures were performed on Linux Ubuntu version 18.04.4 LTS, utilizing a system with NVIDIA GeForce RTX 2070 SUPER and 8-GB V-RAM. Furthermore, the code implementations were carried out using Pytorch 2.4.3 in Python version 3.11.5.

### Network architecture

**Fig. 1:**
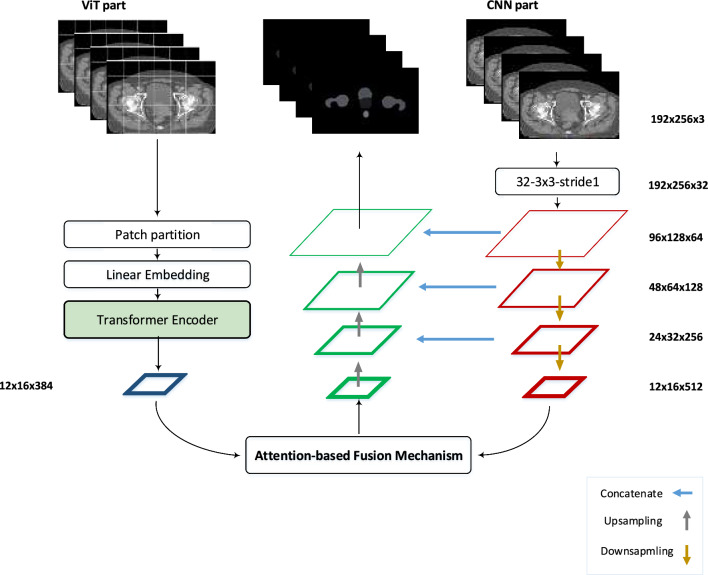
Hybrid CNN-ViT network architecture

We have implemented hybrid CNN-ViT networks that combine convolutional and transformer techniques. Figure 1 illustrates the architecture of the hybrid CNN-ViT networks, which consists of 2 parallel parts: CNN encoding and transformer encoding. These components operate simultaneously to process the input data. Furthermore, there is a fusion part that integrates and combines the outputs of these parallel components.

#### CNN encoding part

The input image passes through a CNN encoder, which extracts local features and captures spatial information. This results in a set of CNN features. The CNN encoder is composed of multiple convolutional layers, pooling layers, and activation functions. In this study, we used two transfer learning network architectures, VGG16-UNet and ResNet50-UNet, for the CNN encoding part.

#### Transformer encoding part

The structure of the transformer part is based on the conventional encoder-decoder architecture. This part begins with global self-attention and gradually restores the local details. The input image is initially divided into patches of equal size. Subsequently, these patches are flattened and forwarded into a linear embedding layer.

#### Fusion block

**Fig. 2:**
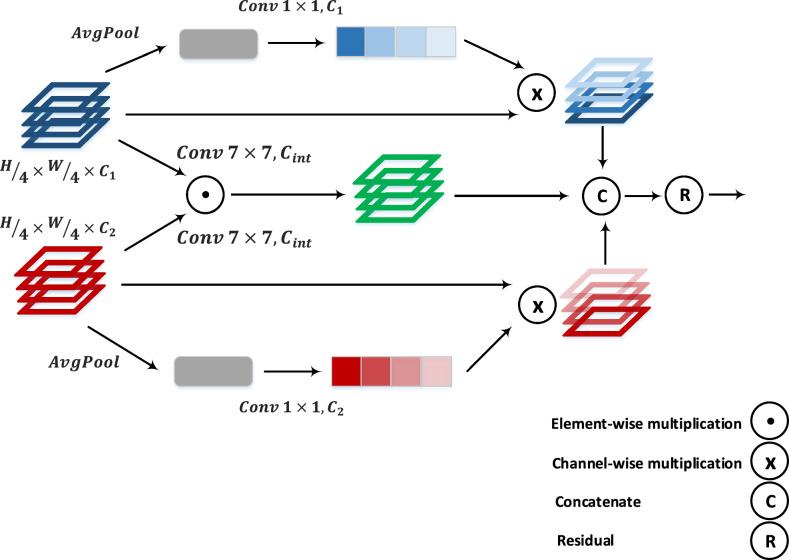
The proposed fusion module

Both parts extract features of the same resolution, which are then inputted into our proposed attention-based fusion module. As illustrated in Fig. 2, the two tensors from the CNN and ViT branches are weighted using a global attention unit and then concatenated by the middle spatial attention branch. By using this technique, the model leverages both global and spatial attention units to fuse the extracted features from both models.

### Model training strategy

We trained our models using transfer learning and fine-tuning on an ImageNet dataset. The ResNet50-UNet-ViT and VGG16-UNet-ViT were trained for 50 epochs using the Adam optimizer (learning rate = 10^−4^) with a batch size of 5.

### Model loss function

We employed a multi-class weighted cross-entropy (CE) intersection-over-union (IOU) loss function to train the networks. The inclusion of class weights in the loss function serves the purpose of adjusting it to penalize false positives or false negatives more significantly

1 $${Loss={Loss}_{IOU}^{w}+Loss}_{CE}^{w}$$

During the training process, the weights vector {w} for each organ is calculated by considering the number of class weights associated with that specific organ.

### Network evaluation criteria

Automatic contours are compared with manual contours in terms of geometry. To evaluate the geometry, we used spatial overlap-based metrics, volume-based metrics, and spatial distance-based metrics.

The DSC is a spatial overlap index that calculates how much of the reference contour and the result contour overlap. Higher DSC results from more overlaps between the result and reference contour.

2 $$Dice\, Coefficient=2*\left(Reference\cap Result)/(Reference+Result\right)$$

Volume-based metrics include volume overlap error and Relative Volume Difference (RVD).

3 $$VOE=\left(1-\left(Reference\cap Result)/(Reference+Result\right)\right)*100$$

RVD calculates the relative difference in volume between the binary objects in the two images. It is calculated according to the following formula.

4 $$RVD=\left(\left(Result-Reference)/(Reference\right)\right)*100$$

Hausdorff distance (HD) is a spatial distance-based metric that indicates the maximum distance of 1 set (automatic contour) to the closest point in another set (manual contour), measured in millimeters. A smaller HD value corresponds to better results. HD can be significantly affected by outlier data, so we used HD95.

The HD between X and Y is:

5 $${\text{HD }}\left( {{\text{X}},{\text{ Y}}} \right) \, = {\text{max }}({\text{hd }}\left( {{\text{Y}},{\text{ X}}} \right),{\text{ hd }}\left( {{\text{X}},{\text{ Y}}} \right))$$

where (hd (Y, X) is the one-sided HD from X to Y that measures the maximum distance from any point in X to its closest neighbor in Y. Mathematically, this is expressed as:

6 $${\text{hd }}\left( {{\text{Y}},{\text{ X}}} \right) \, = ||\mathop {\max }\limits_{{{\text{y}} \in {\text{Y}}}} \mathop { \mathop {min}\limits_{{{\text{x}} \in {\text{X}}}} }\limits_ {\text{ || x}} - {\text{y|| 2}}$$

where, || . || represents the Euclidean distance. We can similarly calculate the one-sided HD from Y to X:

7 $${\text{hd }}\left( {{\text{Y}},{\text{ X}}} \right) \, = \mathop {\max }\limits_{{{\text{y}} \in {\text{Y}}}} \mathop { \mathop {min}\limits_{{{\text{x}} \in {\text{X}}}} }\limits_ {\text{ || x}} - {\text{y || 2}}$$

Another spatial distance-based metric is the average surface distance (ASD), which represents the mean distance between the boundary points of an automatically segmented region and the boundary points of the ground truth.

The ASD is calculated as follows:

8 $$ASD\left( {A,B} \right) = 1/\left| {s(A)} \right| + \left| {S(B)} \right| + \sum\limits_{{S_{A} \in S\left( A \right)}} {d\left( {s_{A} ,S\left( B \right)} \right) + \sum\limits_{{S_{B} \in S\left( B \right)}} {d\left( {s_{B} ,S\left( A \right)} \right)} }$$

where $d\left(v,S\left(A\right)\right)$ is the shortest distance from an arbitrary voxel $v$ to the set of surface voxels $S(A)$and is defined as follows:

9 $$d\left( {v,S\left( A \right)} \right) = \,\mathop {\min \left\| {v\, - \,S_{A} } \right\|}\limits_{{S_{A} \in \,S\left( A \right)}}$$

where, || represents the Euclidean distance.

## Results

### Ablation study

We assessed the performance of models using only CNN components (ablating ViT). Training a purely ViT-based model (ablating CNN) was not feasible due to our hybrid CNN-ViT architecture's reliance on convolutional operations for decoding. To evaluate the impact of a purely ViT approach, we trained an additional model, Swin-UNet [21] (ViT), a fully transformer-based model.

### Quantitative results

We successfully segmented five pelvic organs (prostate, bladder, rectum, and femoral heads) of prostate cancer patients using our proposed 2D hybrid CNN-ViT segmentation networks. To validate their efficacy, we evaluated the hybrid CNN-ViT networks by comparing them with the corresponding pure CNN models. We used the same patient dataset to train and test these network configurations. The CNN models include ResNet50-UNet and VGG16-UNet, which use ResNet50 and VGG16 backbones as CNN encoders. It is noteworthy that all five classes were simultaneously trained using a single network configuration, forward propagation, and loss function.

Table 1 summarizes the quantitative analysis, presenting the mean and standard deviation (SD) of various metrics. We evaluated the impact of ablating each component on the model's performance, as measured by these metrics. As shown in the table, both ResNet50-UNet-ViT and VGG16-UNet-ViT achieve more precise segmentation compared to their corresponding pure convolutional and ViT networks. Furthermore, VGG16-UNet-ViT outperforms ResNet50-UNet-ViT in all five classes.

**Table 1:** Quantitative evaluation of the hybrid CNN-ViT networks compared to the corresponding pure CNN and transformer networks. Negative RVD values indicate a predicted volume smaller than the reference volume, whereas positive RVD values indicate a predicted volume larger than the reference volume

Prediction on the test set							
Organ	Model	DSC (%)	HD95 (mm)	RVD (%)	ASD (mm)	Sensitivity (%)	p-value(DSC)
**Prostate**	ViT	83.91 ± 0.63	11.89 ± 1.65	−16.40 ± 1.42	3.24 ± 1.71	81.04 ± 1.36	< 0.001* compared to ResNet50-UNet-ViT and VGG16-UNet-ViT
	ResNet50-UNet	86.01 ± 1.84	3.11 ± 2.09	−12.22 ± 2.91	1.38 ± 0.71	85.90 ± 1.02	0.014* compared to ResNet50-UNet-ViT
	ResNet50-UNet-ViT	90.02 ± 1.00	2.85 ± 1.46	−6.34 ± 1.78	0.96 ± 0.97	89.55 ± 1.45	
	VGG16-UNet	89.16 ± 1.03	2.44 ± 1.21	** + 0.71 ± 1.44**	0.91 ± 0.81	87.99 ± 0.69	<0.001* compared to VGG16-UNet-ViT
	VGG16-UNet-ViT	**91.75 ± 1.36**	**2.00 ± 1.11**	+ 1.23 ± 1.02	**0.53 ± 0.24**	**91.10 ± 1.00**	
**Bladder**	ViT	91.22 ± 0.94	1.41 ± 0.86	+ 2.37 ± 0.84	0.84 ± 0.69	91.32 ± 0.94	< 0.001* compared to ResNet50-UNet-ViT and 0.001* compared to VGG16-UNet-ViT
	ResNet50-UNet	94.24 ± 1.02	1.51 ± 0.86	+ 1.00 ± 0.79	**0.42 ± 0.84**	93.99 ± 1.84	0.077 compared to ResNet50-UNet-ViT
	ResNet50-UNet-ViT	94.98 ± 0.83	1.32 ± 0.80	+ 2.44 ± 1.37	1.04 ± 0.84	94.67 ± 1.03	
	VGG16-UNet	94.04 ± 1.23	1.89 ± 0.63	+ 2.42 ± 0.78	0.85 ± 0.81	94.24 ± 1.71	0.001* compared to VGG16-UNet-ViT
	VGG16-UNet-ViT	**95.32 ± 0.96**	**1.30 ± 0.99**	** + 0.06 ± 1.01**	0.64 ± 0.61	**95.01 ± 0.87**	
**Rectum**	ViT	80.46 ± 1.09	8.63 ± 1.22	−4.47 ± 0.96	3.49 ± 0.86	80.11 ± 1.23	0.016* compared to ResNet50-UNet-ViT and 0.001* compared to VGG16-UNet-ViT
	ResNet50-UNet	83.56 ± 1.45	4.23 ± 1.46	+ 1.10 ± 0.88	1.00 ± 0.57	83.20 ± 1.32	0.098 compared to ResNet50-UNet-ViT
	ResNet50-UNet-ViT	83.86 ± 1.69	3.25 ± 1.11	−2.03 ± 1.04	0.87 ± 1.01	83.07 ± 0.91	
	VGG16-UNet	85.01 ± 1.22	**3.11 ± 0.89 **	−5.17 ± 1.06	0.63 ± 1.22	84.45 ± 0.90	0.001* compared to VGG16-UNet-ViT
	VGG16-UNet-ViT	**87.00 ± 1.97**	4.46 ± 0.94	**−1.49 ± 1.54**	**0.21 ± 0.88**	**86.52 ± 1.00**	
**RFH**	ViT	94.06 ± 0.63	1.72 ± 1.10	+ 1.30 ± 1.14	0.61 ± 0.82	93.29 ± 0.84	0.031* compared to ResNet50-UNet-ViT and 0.022* compared to VGG16-UNet-ViT
	ResNet50-UNet	95.14 ± 0.97	1.61 ± 0.94	+ 1.88 ± 0.97	**0.27 ± 0.64 **	95.16 ± 1.05	0.011* compared to ResNet50-UNet-ViT
	ResNet50-UNet-ViT	95.83 ± 0.95	**1.22 ± 0.92 **	−1.52 ± 1.42	0.81 ± 0.95	94.90 ± 1.12	
	VGG16-UNet	95.75 ± 0.91	1.36 ± 1.51	+ 1.37 ± 0.84	0.49 ± 0.60	95.09 ± 0.55	0.025* compared to VGG16-UNet-ViT
	VGG16-UNet-ViT	**96.30 ± 0.65**	1.30 ± 1.01	**−1.15 ± 1.25**	0.38 ± 0.68	**96.39 ± 0.69**	
**LFH**	ViT	93.87 ± 1.45	1.89 ± 0.90	+ 1.51 ± 0.81	1.10 ± 0.80	93.39 ± 1.01	0.019* compared to ResNet50-UNet-ViT and 0.004* compared to VGG16-UNet-ViT
	ResNet50-UNet	95.02 ± 1.00	1.40 ± 1.31	−4.01 ± 1.24	**0.39 ± 0.56 **	94.26 ± 0.84	0.147 compared to ResNet50-UNet-ViT
	ResNet50-UNet-ViT	94.80 ± 0.97	1.41 ± 0.86	+ 2.47 ± 0.91	0.51 ± 1.11	93.92 ± 1.24	
	VGG16-UNet	95.72 ± 0.59	1.60 ± 1.04	+ 0.69 ± 0.42	1.03 ± 1.26	95.08 ± 0.86	0.009* compared to VGG16-UNet-ViT
	VGG16-UNet-ViT	**96.34 ± 0.63**	**1.24 ± 1.44**	**−0.72 ± 0.92**	0.49 ± 1.39	**96.22 ± 0.98**	

The best performance for each organ is highlighted in bold

Additionally, we conducted a paired t-test to obtain the p-values to compare the results of the CNN method with our proposed hybrid CNN-ViT segmentation network. The analysis demonstrates that ResNet50-UNet-ViT achieves the highest performance in contouring the prostate, bladder, rectum, and femoral heads compared to ResNet50-UNet with statistical significance. To determine statistical significance, we calculated the P-value by comparing the hybrid CNN-ViT with the corresponding pure CNN model. Statistically significant improvements are indicated with an asterisk (*) when the P-value is less than 0.05.

Table 2 summarizes the impact of ablating in the global attention unit within the fusion module on the model's performance, as measured by DSC. As shown in the table, both ResNet50-UNet-ViT and VGG16-UNet-ViT generally achieve more precise segmentation using a 7 × 7 convolutional kernel compared to other kernel sizes.

**Table 2:** Impact of kernel size of the global attention unit on model performance

Organ	Model	Kernel size	DSC (%)
Prostate	ResNet50-UNet-ViT	Conv 3*3	88.10 ± 0.63
		Conv 5*5	89.42 ± 0.89
		Conv 7*7	**90.02 ± 1.00**
	VGG16-UNet-ViT	Conv 3*3	90.84 ± 1.74
		Conv 5*5	91.05 ± 1.22
		Conv 7*7	**91.75 ± 1.36**
Bladder	ResNet50-UNet-ViT	Conv 3*3	94.22 ± 0.63
		Conv 5*5	**95.04 ± 1.21**
		Conv 7*7	94.98 ± 0.83
	VGG16-UNet-ViT	Conv 3*3	91.46 ± 1.36
		Conv 5*5	91.36 ± 1.00
		Conv 7*7	**95.32 ± 0.96**
Rectum	ResNet50-UNet-ViT	Conv 3*3	83.84 ± 1.21
		Conv 5*5	**84.26 ± 0.72**
		Conv 7*7	83.86 ± 1.69
	VGG16-UNet-ViT	Conv 3*3	84.11 ± 0.81
		Conv 5*5	86.28 ± 1.21
		Conv 7*7	**87.00 ± 1.97**
RFH	ResNet50-UNet-ViT	Conv 3*3	95.49 ± 0.85
		Conv 5*5	94.87 ± 1.00
		Conv 7*7	**95.83 ± 0.95**
	VGG16-UNet-ViT	Conv 3*3	94.11 ± 0.65
		Conv 5*5	96.04 ± 1.24
		Conv 7*7	**96.30 ± 0.65**
LFH	ResNet50-UNet-ViT	Conv 3*3	**95.04 ± 0.81**
		Conv 5*5	94.29 ± 0.77
		Conv 7*7	94.80 ± 0.97
	VGG16-UNet-ViT	Conv 3*3	95.07 ± 0.59
		Conv 5*5	95.27 ± 1.16
		Conv 7*7	**96.34 ± 0.63**

The best performance for each organ is highlighted in bold

### Qualitative results

**Fig. 3:**
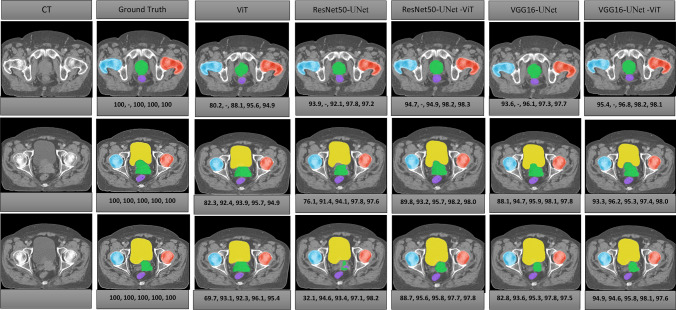
The overlay segmentation of the prostate (green), bladder (yellow), rectum (purple), RFH (red), and LFH (blue) achieved by ViT, ResNet50-UNet, ResNet50-UNet-ViT, VGG16-UNet, and VGG16-UNet-ViT segmentation networks are demonstrated in the axial view. The DSC values of the prostate, bladder, rectum, RFH, and LFH are represented by the quintuple array at the bottom of each image

The predicted contours of the five classes for the five networks are presented in Fig. 3. The contours produced by the hybrid CNN-ViT segmentation networks exhibit a high degree of similarity to the ground truth contours.

**Fig. 4:**
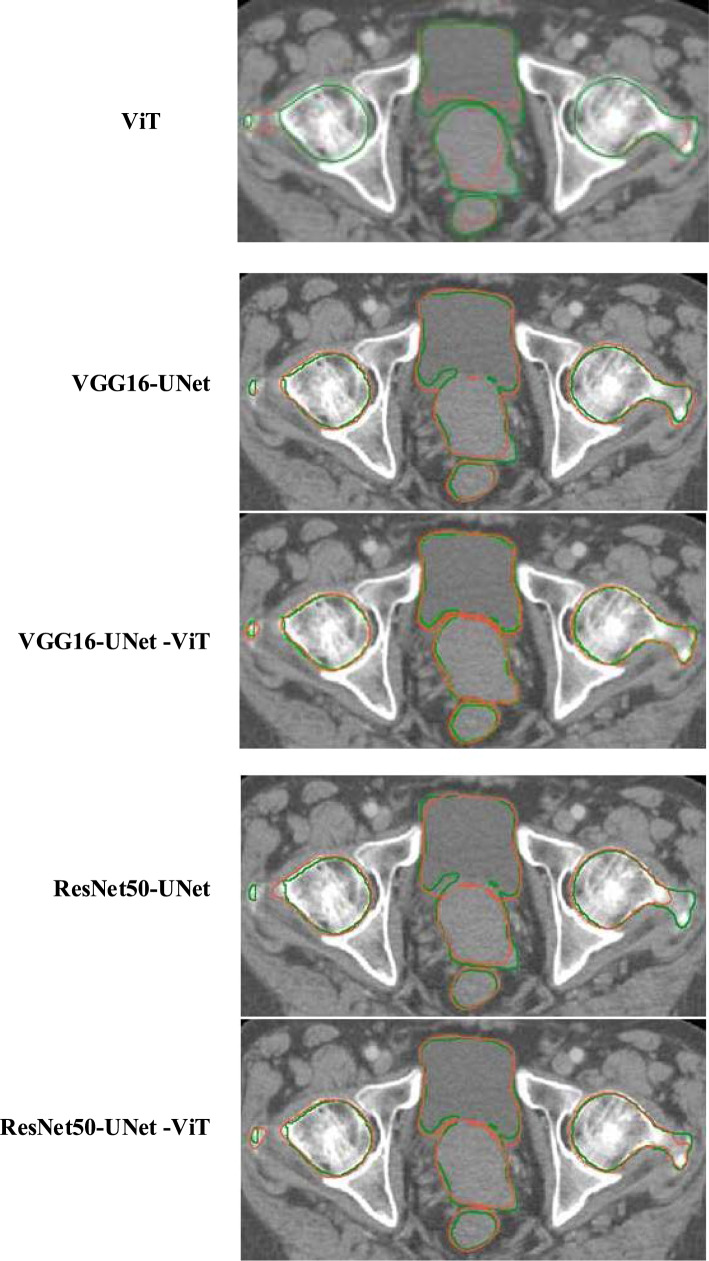
The overlay segmentation of the reference (green) and automated contours (red)

Figure 4 displays the reference organ boundaries and segmentation results of a randomly selected slice from the testing dataset. It is evident that our suggested approach accurately contours the organ boundaries, as indicated by the significant overlap between the automated and reference segmentation outcomes.

### Comparison with the state-of-the-art techniques

Table 3 provides a comparison between our proposed method's performance and other state-of-the-art methods in the literature.

**Table 3:** Comparison of the present study with the state-of-the-art studies (‘-’ denotes that the metric is not reported)

Study	Method	Sample size	Organ	DSC (%)	ASD (mm)	Sensitivity (%)
Kazemifar et al. [8] 2018	2D U-Net	85	Prostate	88.2	0.4	87.0
			Bladder	95.4	0.4	95.0
			Rectum	92.6	0.2	92.0
He et al. [7] 2019	FCN	313	Prostate	89.0	1.3	88.0
			Bladder	94.0	0.9	93.0
			Rectum	89.0	1.3	90.0
Wang et al. [12] 2019	FCN	313	Prostate	89.0	1.3	85.0
			Bladder	94.0	1.2	93.0
			Rectum	89.0	1.5	88.0
Kiljunen et al. [25] 2020	3D U-Net	900	Prostate	82.0	3.3	–
			Bladder	93.0	6.1	–
			Rectum	84.0	11.4	–
			RFH	69.0	24.7	–
			LFH	68.0	25.0	–
He et al. [11] 2021	MetricUNet-HCP	339	Prostate	88.4	1.4	88.0
Zhang et al. [22] 2021	ARPM-Net	120	Prostate	88.0	1.6	–
			Bladder	97.0	1.9	–
			Rectum	86.0	3.1	–
			RFH	97.0	1.8	–
			LFH	97.0	1.9	–
Pan et al. [13] 2022	Token-based transformer network	94	Prostate	84.0	2.03	84.0
			Bladder	94.0	1.06	95.0
			Rectum	89.0	1.30	89.0
			RFH	95.0	0.91	95.0
			LFH	95.0	0.96	95.0
Shen et al. [15] 2023	CUNet	217	Prostate	84.0	–	–
			Bladder	91.3	–	–
			Rectum	78.3	–	–
			RFH	89.7	–	–
			LFH	89.9	–	–
Mofid et al. [16] 2024	3D nnU-net	118	Prostate	82.0	–	–
			Bladder	97.0	–	–
			Rectum	90.0	–	–
			RFH	96.0	–	–
			LFH	96.0	–	–
Our study	VGG16-UNet-ViT	104	Prostate	91.7	0.5	91.1
			Bladder	95.3	0.6	95.0
			Rectum	87.0	0.2	86.5
			RFH	96.3	0.4	96.4
			LFH	96.3	0.5	96.2

## Discussion

In this study, our objective was to investigate automated male pelvic multi-organ contouring from multi-center and diverse planning CT images using hybrid CNN-ViT networks that combine convolution and transformer techniques. We introduced a novel attention-based fusion module that merges the detailed features extracted through convolution with the global features obtained through the transformer.

Experiments conducted on multicenter planning CT images indicate that combining the ViT structure with the CNN network resulted in superior performance for all organs compared to pure CNN and transformer architectures, except for the LFH in the ResNet50-UNet network. As evidenced by the p-values reported in Table 1, VGG16-UNet-ViT demonstrated statistically superior accuracy compared to VGG16-UNet and ViT for all structures in terms of DSC.

According to Table 3, our DSC for the prostate was superior compared to other similar studies. This superiority can be primarily attributed to the utilization of a combination of convolution and transformer techniques.

In our proposed method, the mean DSC for the bladder is 95.54%, which ranks second after the study of Zhang et al. [22]. Although they achieved higher DSCs for the bladder (97%), their study's reliance on a single observer and a single CT device as a reference introduces potential bias in the results, particularly when compared to a multicenter study.

In our study, the DSC for the rectum is 86.8%, which is comparatively lower than the results reported in certain similar studies. Among similar studies, Kazemifar et al.’s method [10] achieved the best segmentation result for the rectum. Our study is not directly comparable to their study for the rectum because they used patients with endo-rectal balloon insertion. Endo-rectal balloons are commonly used in the radiotherapy of prostate cancer patients to spare the rectum [23].

Our findings, which are based on the utilization of private and diverse datasets, are consistent with the results of studies conducted by Kazemifar et al. [8]; He et al. [11]; Zhang et al. [22]; Kearney et al. [24]; and Wang et al [12]. All of these methods obtained satisfactory results for RFH, LFH, and bladder. RFH and LFH contouring, due to their high contrast, is easy for networks [25]. Similarly, delineating bladder boundaries is relatively easy due to its distinct wall structure and large size [12]. However, accurately delineating the boundaries of the prostate and rectum presents more significant challenges due to their smaller size and lower contrast [7], especially in regions where these two organs are in close proximity.

Sensitivity is a commonly used metric in image analysis [12]. The hybrid CNN-ViT networks exhibit superior sensitivity compared to the corresponding pure CNN networks.

We evaluated the models using different metrics (spatial overlap-based metrics, volume-based metrics, and spatial distance-based metrics), as shown in Table 1, to ensure result consistency. In general, the hybrid CNN-ViT networks exhibit lower HD95 and ASD values compared to the corresponding pure CNN networks. As expected, the rectum demonstrates the highest RVD (-1.84%) among all structures in the VGG16-UNet-ViT network. This observation is consistent with its lower DSC value (86.8%). This means that the predicted volume is 1.84% smaller than the reference volume. This small volume difference is likely to have a negligible effect on the dose-volume metrics used in routine radiotherapy treatment planning optimization.

VGG16-UNet-ViT for all organs and ResNet50-UNet-ViT, except for the LFH and rectum, achieve more precise segmentation using a 7 × 7 convolutional kernel compared to other convolution sizes. This improved performance with a 7 × 7 kernel is likely attributed to its larger receptive field and better ability to capture contextual information.

## Conclusion and future work

This paper introduces a segmentation network that uses a novel attention-based fusion method to combine the ViT and CNN architectures for male pelvic multi-organ contouring on planning CT images. Our findings demonstrate that integrating convolutional and transformer techniques resulted in superior segmentation performance compared to solely relying on either convolutional or transformer networks.

Additionally, the proposed method achieves more precise contours compared to state-of-the-art techniques. The results show promise as a reliable and efficient tool to aid in prostate radiotherapy treatment planning. Automatic contouring is a valuable tool in radiotherapy treatment planning; however, it cannot be solely relied upon as the definitive treatment contours. It is imperative that a qualified physician evaluates the contours and makes any required modifications to ensure accuracy and precision. Incorporating automated contouring methods in clinics provides several benefits, such as minimizing variability between different observers and accelerating the segmentation process.

Our work has certain limitations. We used a limited test set consisting of only 20 cases, which may not fully represent the diverse range of male pelvic CT images. To address this, we plan to validate our proposed method on a larger dataset to demonstrate its applicability and generalizability. Additionally, in the future, we aim to investigate the dosimetry impact of deep learning-based auto-contoured structures compared to manual contours for radiotherapy treatment planning.

## Data Availability

The datasets used and analyzed in the study are available from the corresponding author upon reasonable request.

## Abbreviations

CT: Computed Tomography
CNN-ViT: Convolutional Neural Network-Vision Transformer
CNN: Convolutional Neural Network
ViT: Vision Transformer
CTV: Clinical Target Volume
OAR: Organs At Risk
DSC: Dice Similarity Coefficient
RFH: Right Femoral Head
LFH: Left femoral head
SD: Standard deviation
CE: Cross-Entropy
IOU: Intersection-Over-Union
VOE: Volume Overlap Error
HD: Hausdorff Distance
ASD: Average Surface Distance
